# The Way We See Others in Intercultural Relations: The Role of Stereotypes in the Acculturation Preferences of Spanish and Moroccan-Origin Adolescents

**DOI:** 10.3389/fpsyg.2020.610644

**Published:** 2021-01-11

**Authors:** Ana Urbiola, Lucía López-Rodríguez, María Sánchez-Castelló, Marisol Navas, Isabel Cuadrado

**Affiliations:** ^1^Center for the Study of Migration and Intercultural Relations, University of Almería, Almeria, Spain; ^2^Department of Psychology, University of Almería, Almería, Spain

**Keywords:** stereotypes, immorality, morality, acculturation preferences, acculturation perceptions, identity, adolescents, Moroccan immigrants

## Abstract

Although the relationship between stereotypes and acculturation preferences has been previously studied from the majority perspective among adults, the perspective of adolescents and minority groups is understudied. This research analyzed the contribution of four stereotype dimensions (i.e., morality, immorality, sociability, and competence) to the acculturation preferences of Spanish adolescents and adolescents of Moroccan-origin, the moderating role of stereotypes in intergroup acculturation discrepancies, and the interaction of stereotypes with acculturation perceptions on acculturation preferences. A sample of 488 Spanish adolescents and 360 adolescents of Moroccan-origin living in Spain, from 12 to 19 years old, reported how moral, immoral, social, and competent they perceive each other to be. Spanish adolescents reported their perception about how Moroccan youth were acculturating in terms of maintaining their original culture and adopting the host culture, and their acculturation preferences in the same dimensions. Adolescents of Moroccan-origin reported to what extent they were maintaining their original culture and adopting the host culture, their acculturation preferences, and their ethnic and national (Spanish) identity. Results showed that adolescents of Moroccan-origin reported more positive perceptions of Spanish youth than conversely. The perceived immorality of the outgroup was important for understanding the preferences for adopting the host culture of both groups, but in the opposite direction. The four stereotype dimensions modulated the majority-minority discrepancies in preferences for cultural adoption. An analysis of the interaction between stereotypes and perceived adoption on acculturation preferences showed that when Spanish adolescents perceived that Moroccan youth were not adopting the Spanish culture, perceived morality and sociability played a role in their preferences for adoption. The less moral and sociable Moroccans were perceived, the more preference for cultural adoption. These findings support the importance of considering stereotypes in acculturation studies of majority and minority groups, as well as the relevance of including these perceptions in interventions aimed at improving intercultural relations.

## Introduction

The phenomenon of migration has significant consequences on human life. One of the most important forms of consequence is the continuous contact with members of other cultures, triggering noteworthy changes in both the immigrants and the host society (Redfield et al., 1936). These changes are conceptualized in the psychosocial literature as the acculturation process. Berry (2005) defines acculturation as “the dual process of cultural and psychological change that takes place as a result of contact between two or more cultural groups and their individual members” (p. 698).

Within Europe, Spain has become an important recipient of immigration in the last two decades. Of the foreign residents in this country, 15.2% are under the age of 16 years. Acculturation preferences of adolescents from both the majority and minority groups living in the same context can settle their future peaceful vs. conflictual relationships, and contribute – in the long term – to social inclusion and well-being in contemporary societies. We focus on adolescence because of two main reasons. Firstly, because the impact of acculturation processes and the identity development considering both the original and the new host culture are specifically complex and sensitive during adolescence. The relationship between acculturation and identity (ethnic and national) has been claimed in several works (Phinney, 2003; Schwartz et al., 2006). The relevance of identity and identification processes takes more importance in adolescence, and even more for immigrant adolescents (Schwartz et al., 2006). They have to face the challenge of constructing or endorsing an identity that incorporates elements of both the cultural origins and the receiving cultures, in addition to the difficulties of constructing an identity that characterizes this developmental period (Schwartz et al., 2006). Secondly, because at this developmental stage, stereotypes are still flexible and relatively easy to change. Therefore, mapping the relations between stereotypes toward outgroups and intergroup behavior in intercultural encounters is essential to facilitate the change of stereotypes and biased intergroup perceptions at this stage before they get firmly established in adulthood, increasing intergroup hostility (Constantin and Cuadrado, 2019).

Given the importance of acculturation preferences for cultural understanding, several researchers have turned to the study of those variables associated with more flexible acculturation preferences that facilitate cultural negotiation and positive coexistence. Among them, the way we see others – that is, intergroup stereotypes – constitute basic dimensions in this process of mutual understanding. Despite the importance of social beliefs about groups in the acculturation process, the evidence of the relationship between stereotypes and acculturation preferences is scarce, especially among adolescents and considering the minority perspective. Although this relationship has been established among adults, the way stereotypes can interact with other variables to predict acculturation preferences or how stereotypes contribute to modulate discrepancies in acculturation preferences between majority and minority groups remain unexplored.

Accordingly, the main question that motivated this inquiry was to understand the specific role that distinct stereotype dimensions play in the acculturation preferences of Spanish adolescents and adolescents of Moroccan-origin living in Spain. Moroccans constitute the largest immigrant group in this country (15.9% of foreign residents; INE, 2020) and systematically receive worse evaluations from Spaniards compared to members of other immigrant groups, such as Romanians or Ecuadorians (e.g., Navas et al., 2012; López-Rodríguez et al., 2013, 2016). We first follow a descriptive approach by analyzing mutual stereotypes (Objective 1), and the majority-minority discrepancies in adolescents’ acculturation perceptions and preferences (Objective 2). Then, we analyze the specific contribution of four different stereotype dimensions (i.e., morality, immorality, sociability, and competence), beyond traditional variables of acculturation perceptions (for majority and minority groups) and national and ethnic identities (for the minority group), to predict their acculturation preferences (Objective 3). Based on the diagnostic value of (im)morality, we anticipate that these specific dimensions will be more relevant than the other stereotype dimensions to adolescents’ acculturation preferences. This research also tests if stereotypes modulate the discrepancies between Spanish adolescents and adolescents of Moroccan-origin in their acculturation preferences (Objective 4). Finally, we address an innovative question by analyzing whether stereotypes might be more predictive of acculturation preferences depending on acculturation perceptions (Objective 5). As far as we know, no previous research has covered this gap in the theory of acculturation and stereotypes. Coherent to the function of stereotypes as cues to orientate rapid decisions and social judgments, we hypothesize that stereotypes have no isolated effects on acculturation preferences, but they combined with other important factors such as acculturation perceptions. According to this reasoning, stereotypes might have a more prominent role in acculturation preferences under more threatening circumstances, that is, when majority members perceive immigrant-origin adolescents are not adopting the host culture.

### The Complex Process of Acculturation

According to the extremely influential acculturation model developed by Berry (1997), immigrants must face two fundamental questions when living in the host country: (1) To what extent is it important to maintain my cultural heritage? and (2) To what extent is it important to establish relations with the host society and adopt the host culture? Regarding identity, these two questions refer to maintaining a sense of belonging to one’s ethnic community (ethnic identity), and to developing a sense of host national belonging (national identity), respectively (Zhang et al., 2018). Initially considered orthogonal, these two dimensions were combined to yield four acculturation categories. Nowadays, however, several researchers prefer to measure these underlying dimensions of maintenance and contact/adoption due to methodological considerations (Rudmin, 2003), and to avoid losing precious information (Brown and Zagefka, 2011).

Although acculturation models were initially focused on the acculturation preferences of minority groups, the importance of examining also the preferences of the majority group was subsequently recognized (Zagefka et al., 2014), given its substantial influence and power in the host society (Geschke et al., 2010). Accordingly, in the last decades, considerable work has focused on the majority perspective (e.g., Bourhis et al., 1997; Piontkowski et al., 2000; Zagefka et al., 2007). Different models recognize the interactive nature of acculturation (for a review see Horenczyk et al., 2013) such as the mutual acculturation model (Berry, 2006), the interactive acculturation model (IAM; Bourhis et al., 1997), the concordance model of acculturation (Piontkowski et al., 2002), or the relative acculturation extended model (RAEM; Navas et al., 2005, 2007). In the present research, we use the RAEM to capture the mutuality in acculturation processes.

Similar to previous interactive models, the RAEM takes into account two acculturation dimensions (maintenance of original culture and adoption of host culture) and considers both the majority and minority perspectives in order to understand better the acculturation processes. Moreover, this model differentiates between the real and the ideal plane in the acculturation process. The real plane refers to the acculturation strategies that minority groups declare to practice and the perception of majority members about these strategies. The ideal plane refers to the acculturation preferences (that is, what both minority members and hosts would prefer if they could choose, in terms of cultural adoption and maintenance for minority members). Moreover, the RAEM considers simultaneously different domains as a way to capture the inherent complexity of the acculturation process, asking for different areas of socio-cultural reality (e.g., political, work, social well-being, and consumer habits) or others more central to the cultural process (e.g., social relations, family relations, religion, and values).

This model gives particular importance to the acculturation perceptions and preferences of both minority and majority members. This is especially relevant from a psychosocial perspective given that the analysis and potential interventions on biased perceptions and discrepant attitudes take a central stage on intercultural conflict resolution. When comparing majority and minority perspectives, acculturation discrepancies can be found. According to Bourhis et al. (1997), when the profile of acculturation orientations obtained for the host community and the immigrant group match very little or not at all, acculturation discordance between both communities can be problematic or conflictual, respectively. These discrepancies are usual in acculturation research, with immigrants usually preferring to adopt the host culture less and maintaining the original culture more than what the members of the host country prefer they do (e.g., in Spain, Navas et al., 2007; Navas and Rojas, 2010). In the specific case of adolescents, a research conducted in two Mediterranean countries (Italy and Spain) found that there was little consensus between immigrant and host adolescents in their acculturation preferences (López-Rodríguez et al., 2014a; Mancini et al., 2018): immigrant adolescents living in both countries preferred to maintain their original culture more than what host adolescents preferred. Additionally, young immigrants in Italy preferred to adopt the host culture to a lower extent compared to what their Italian national peers demanded. This discrepancy in preference for adoption was inverted in Spain, with young immigrants preferring to adopt more the host culture compared to what Spanish adolescents demanded (especially in school or consumer habits). This study, however, included immigrants from different backgrounds. As acculturation is context-depended, results might vary when a specific cultural background is considered.

The study of majority members’ acculturation preferences is essential because host majority members can influence the acculturation strategies of minority members, “who in turn may also affect the orientations of the host majority” (Bourhis et al., 1997, p. 375). As Van Acker and Vanbeselaere (2011) have claimed, knowing the antecedents of majority members’ preferences regarding the acculturation of different minorities offers chances to modify and intervene on majority members’ preferences and to fill the gap between the positions of both the majority and the minority groups, and consequently, to improve intergroup relations. This research analyzes the contribution of intergroup stereotypes beyond traditional variables of acculturation perceptions to predict acculturation preferences among Spanish adolescents and adolescents of Moroccan-origin living in Spain, the moderating role of stereotypes in intergroup acculturation discrepancies, as well as the interaction between stereotypes and acculturation perceptions on acculturation preferences.

### Intergroup Stereotypes and the Acculturation Process

Acculturation processes are characterized by a remarkable complexity and are contingent to the context of intergroup relations. In this line, studies have related majority and/or minority acculturation preferences to different psychosocial variables, such as prejudice, stereotypes, or perceived discrimination (e.g., Maisonneuve and Testé, 2007; López-Rodríguez et al., 2014b; Rojas et al., 2014; Zagefka et al., 2014; López-Rodríguez and Zagefka, 2015; Cuadrado et al., 2017, 2018). The way we see others is essential to understanding intercultural relations. Stereotypes serve as social keys to guide judgments in complex situations as those involved in intercultural relations. With not much social information, stereotypes fulfill the gaps and shape interpretations of different events, being essential in intercultural relations. From the majority perspective, more flexible acculturation orientations such as integration are more strongly preferred for “valued” than “devalued” immigrants, whereas less tolerant acculturation orientations such as assimilation or exclusion are more endorsed for “devalued” than “valued” immigrants (Montreuil and Bourhis, 2001). As far as we know, no previous research has tested whether intergroup stereotypes could modulate acculturation discrepancies between the minority and majority groups.

The stereotype content model (SCM; Fiske et al., 2002) postulates that social group members are evaluated on two basic dimensions of social perception: warmth and competence. The warmth dimension helps to anticipate the intentions of others and includes characteristics such as being sincere or friendly. The dimension of competence allows knowing the capacity of others to achieve their intentions or objectives and includes characteristics such as being intelligent or skillful. Afterward, Leach et al. (2007) demonstrated that warmth consists of two differentiated evaluative components: sociability and morality. According to Brambilla and Leach (2014, p. 398), “sociability pertains to being benevolent to people in ways that facilitate affective relations with them (e.g., friendliness, likeability, and kindness), morality refers to being benevolent to people in ways that facilitate correct and principled relations with them (e.g., honesty, trustworthiness, and sincerity).” Several studies have highlighted the primary and distinctive role of morality on social judgments, impression formation, or in-group and out-group evaluations and reactions (e.g., Brambilla et al., 2011, 2012; López-Rodríguez et al., 2013; Leach et al., 2015; Landy et al., 2016; Cuadrado et al., 2020; see Brambilla and Leach, 2014, for a review). Therefore, there is enough evidence on the importance of analyzing morality and sociability as two separate dimensions of warmth. Studies carried out in Spain with the three-dimensional stereotype content model in adult population confirm the distinctive role of morality (vs. sociability and competence) in outgroup evaluations. These studies have analyzed the perceptions of the majority group toward immigrants from different origins (López-Rodríguez et al., 2013), the perceptions of the minority group toward the majority group (Cuadrado et al., 2017, 2020 Study 2), and the intra-minority perspective where immigrants evaluated other immigrant groups (Cuadrado et al., 2016). Constantin and Cuadrado (2020) have confirmed the superiority of the three-factor model of stereotype content compared to the bi-dimensional one in a study with Spanish adolescents evaluating to Moroccan and Ecuadorian immigrants.

That said, recent studies (e.g., Goodwin and Darley, 2012; Sayans-Jiménez et al., 2017) have shown the importance of also considering the negative aspects of morality (i.e., immorality) in impressions formation and out-group evaluations. According to Brambilla and Leach (2014, p. 400), “when people search for the most diagnostic information available about a person, they search for negative information about that person’s morality.” The negative pole of morality has more evaluative weight than the positive pole. Attributes associated with badness in negative moral actions (e.g., theft, cheating) are more reliable and objective than those related to the goodness of positive moral actions (e.g., donate money; Goodwin and Darley, 2012). The cue-diagnosticity of negative traits related to morality in the impression-formation process is well-known. Skowronski and Carlston (1987) found “that negative behaviors are perceived as more diagnostic than positive behaviors when the former are morality related (honesty-dishonesty)” (p. 689). The negativity effect of morality has motivational, affective and cognitive bases (for a review see Rusconi et al., 2020). Additionally, the methodological research conducted by Sayans-Jiménez et al. (2017) indicated that the addition of negative items of morality (i.e., immorality) allows researchers to explain a bigger amount of variance than when only positive items are used.

Perceived morality and immorality allow us to infer the potential benefits and/or threats that other people or groups represent to our well-being or that of our group (Brambilla et al., 2011, 2012). Therefore, these are prominent stereotypical dimensions in the search for information, impressions formation, and out-group evaluation that must be taken into account. To our knowledge, no studies have yet been conducted using the four dimensional model of stereotype content (i.e., morality, immorality, sociability, and competence) in adolescents from the minority perspective and its relationship with acculturation preferences.

The relationship between these stereotype dimensions and acculturation preferences has received scarce attention in the literature. There are some exceptions. López-Rodríguez et al. (2014b) found that stereotypes (and the perceived threat associated to them) mediated the relationship between perceived adoption and preference for maintenance and adoption of a sample of Spaniards regarding Moroccan immigrants. In this case, positive evaluations were associated to more preference for culture maintenance and less preference for culture adoption *via* perceived threat. When stereotype dimensions were experimentally manipulated, the dimension of morality was the only dimension that affected the desire of majority members for minority group’s maintenance of the original culture (López-Rodríguez and Zagefka, 2015). From the minority perspective, Cuadrado et al. (2017) found that, especially perceived morality, but also perceived competence, indirectly and positively predicted the preference of immigrants for adopting Spanish customs through positive emotions toward Spaniards. These studies have not simultaneously considered the majority and minority perspective, the real (perceptions) and ideal plane (preferences) in the acculturation process, and they have only taken into account three (but not four) stereotype dimensions, ignoring the role of perceived immorality, the most threatening stereotype dimension. Moreover, previous research has not inquired about under which circumstances stereotypes might be more closely related to acculturation preferences. Kosic and Phalet (2006) found a significant interaction effect between perceived maintenance of the Moroccan culture of origin and prejudice on overcategorization. This finding suggests that the interaction between high prejudice and high perceived maintenance is associated with a highly threatening situation displayed as an overcategorization of photographs as Moroccans. Therefore, although prejudice has been found to interact with acculturation perceptions, there is no evidence that stereotypes interact with these perceptions to predict acculturation preferences.

### Objectives and Hypotheses

The main question that motivated this inquiry was to understand the role that distinct stereotype dimensions play in the acculturation preferences of Spanish adolescents and adolescents of Moroccan-origin living in Spain. To achieve this goal, we first described the participants’ perceptions and preferences. Specifically, we analyze the majority-minority discrepancies in adolescents’ acculturation perceptions and preferences, and their mutual stereotypes. We expect Spanish adolescents to have a more negative perception of Moroccan youth than vice versa (H1). Based on previous findings with minority and majority samples of adolescents (e.g., López-Rodríguez et al., 2014a; Mancini et al., 2018), we expect that adolescents of Moroccan origin will report adopting more and maintaining less than what Spanish adolescents perceive them to do (H2a), and prefer to adopt less and maintain more than what host adolescents demand (H2b).

Then, we analyzed the specific contribution of four different stereotype dimensions (i.e., morality, immorality, sociability, and competence), beyond traditional variables of acculturation perceptions (for majority and minority groups) and national and ethnic identity (for the minority group), to predict their acculturation preferences. The main categories of identification considered as important variables in acculturation processes are the ethnic and national categories. Because of that, we include also these categories of identity as classical predictors of acculturation preferences for the minority group. We hypothesize that dimensions of stereotypes will predict acculturation preferences beyond acculturation perceptions (for majority and minority groups) and identity (for the minority group). Based on the diagnostic value of (im)morality (e.g., Skowronski and Carlston, 1987; Sayans-Jiménez et al., 2017), we expect that these two dimensions will be more relevant to adolescents’ acculturation preferences than the other dimensions of stereotypes (H3).

This research also tests whether stereotypes could modulate the discrepancies between Spanish adolescents and adolescents of Moroccan-origin in their acculturation preferences. Previous research has found that less tolerant acculturation orientations such as assimilation or exclusion are more endorsed for “devalued” immigrants toward whom there are negative stereotypes (Montreuil and Bourhis, 2001). We might infer that mutual negative stereotypes would harden acculturation discrepancies, whereas positive mutual perceptions might soften such discrepancies (H4).

Finally, we address an innovative question by analyzing under which circumstances stereotypes might be more predictive of acculturation preferences. Previous research has shown that acculturation perceptions can interact with prejudice (Kosic and Phalet, 2006) as well as the central role that perceived adoption (compared to perceived maintenance) can play in acculturation preferences (López-Rodríguez et al., 2014b). This innovative exploration is relevant since it connects the literature of acculturation and intergroup relations in an interactive way instead of studying the predictive role of stereotypes or acculturation perceptions in isolation, which is likely to often oversimplify people’s psychosocial realities. We hypothesize that stereotypes would play a major role in majority members’ acculturation preferences when they perceived that Moroccan youth were not adopting the Spanish culture because it is a more threatening situation than when minority group members are adopting the host culture (H5).

## Materials and Methods

### Participants

A sample of 488 Spanish adolescents (*M_age_* = 14.79, *SD_age_* = 1.23; 52.4% girls) and 360 adolescents of Moroccan origin living in Spain (*M_age_* = 15.16, *SD_age_* = 1.36; 58.7% girls) from 12 to 19 years old volunteered to participate in this study. They were enrolled in different public secondary schools in Spain. The 36.4% of adolescents of Moroccan origin were born in Spain and 62.2% in Morocco. Most of their parents (97.8% of fathers and 98.6% of mothers) were born in Morocco. For participants who were born in Morocco, the average age of arrival to Spain was 4.84 years (*SD* = 4.39).

### Variables and Instruments

Participants answered one of two similar versions of a questionnaire, changing the out-group evaluated. Spanish adolescents answered the questionnaire assessing Moroccan youth, and adolescents of Moroccan origin assessed Spanish youth. The questionnaires contained instruments to measure the following variables. All items were measured on a five-point Likert scale ranging from 1 (*not at all*) to 5 (*very much*).

#### Stereotypes

This variable was measured through a 17-item scale (from López-Rodríguez et al., 2013; Sayans-Jiménez et al., 2017). The scale consisted of four subdimensions: morality (four items: honest, trustworthy, sincere, and respectful), immorality (five items: aggressive, malicious, harmful, treacherous, and false), sociability (four items: friendly, warm, likeable, and kind) and competence (four items: intelligent, skilful, competent, and efficient). Participants were asked to what extent each adjective described the out-group (Spaniards or Moroccans). The estimated reliability coefficients (Cronbach’s alpha and split-half with Spearman-Brown correction) are included in Table 1.

**Figure 1:**
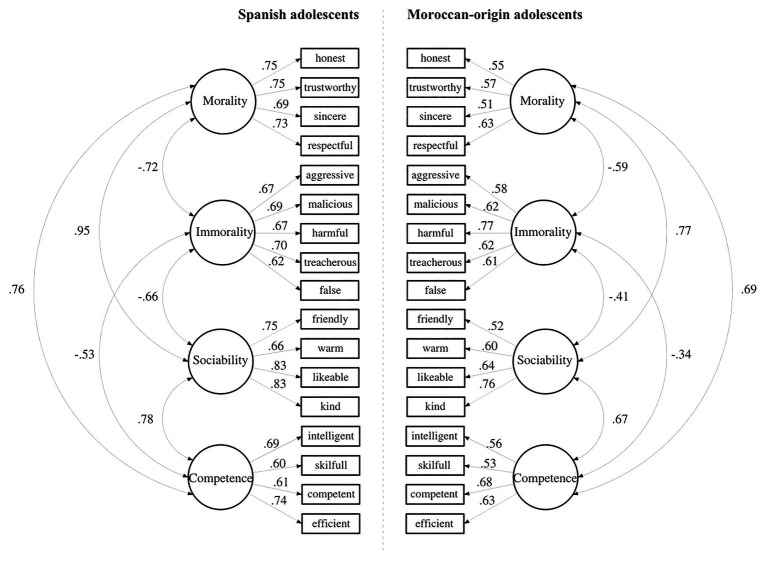
Results of confirmatory factor analysis for the four-factor model for Spanish and Moroccan-origin adolescents. All factor loadings are standardized and statistically significant.

**Table 1:** Estimated reliability coefficients.

	Cronbach’s alpha		Split-Half (Spearman-Brown)	
	SA	MA	SA	MA
Maintenance	0.58	0.68	0.65	0.67
Adoption	0.71	0.66	0.81	0.72
Preferences for maintenance	0.83	0.83	0.86	0.84
Preferences for adoption	0.86	0.77	0.89	0.82
Morality	0.82	0.65	0.84	0.69
Immorality	0.80	0.77	0.74	0.73
Sociability	0.85	0.72	0.86	0.71
Competence	0.76	0.68	0.76	0.63

SA, Spanish adolescents; MA, Moroccan-origin adolescents.

#### Acculturation Perceptions

They were measured through two scales adapted from the RAEM to adolescent populations (López-Rodríguez et al., 2014a; Mancini and Bottura, 2014; Mancini et al., 2018): one for maintaining the customs of origin and another for adopting Spanish customs. Spanish adolescents indicated to what extent they perceived that Moroccan youth maintained the origin customs and to what extent they perceived they have adopted or practiced Spanish customs across six domains (academic, economic, social, family, religion, and values). One example of an item is the following: “To what extent do you think that Moroccan youth living in Spain maintain nowadays the customs of their country in social relations (ways of socializing, usual places for socializing, the way in which they spend their free time, ways of having fun, etc.)?” Adolescents of Moroccan origin indicated to what extent they maintained the Moroccan customs (of their parents’ country of origin) and adopted or practiced Spanish customs in the same domains. One example of an item is the following: “To what extend do you maintain nowadays the Moroccan customs in your family relationships (way of relating to their parents, with older people in the family, with their brothers and sisters, tasks that each member of the family does, etc.)?” Average scores on each scale (cultural maintenance and adoption) were obtained. The estimated reliability coefficients (Cronbach’s alpha and split-half with Spearman-Brown correction) are included in Table 1.

#### Acculturation Preferences

Participants’ preferences were also measured using two scales: one for maintaining the origin customs and another for the adoption of Spanish customs. Spanish adolescents were asked to what extent they would like Moroccan youth to maintain the origin customs and to adopt the Spanish customs. One example of an item is: “If you could choose, to what extent would you like Moroccan youth living in Spain to adopt or practice the Spanish customs in social relations (ways of socializing, usual places for socializing, the way in which they spend their free time, ways of having fun, etc.)?” Adolescents of Moroccan origin were asked to what extent they would like to maintain the Moroccan customs (of their parents’ country of origin) and to adopt the Spanish customs (the items were the same as for Spanish adolescents but asking “To what extent would you like to adopt or practice the Spanish customs?”). Each scale contains six items referring to the same RAEM domains. Average scores on each scale (cultural maintenance and adoption) were obtained. The estimated reliability coefficients (Cronbach’s alpha and split-half with Spearman-Brown correction) are included in Table 1.

#### Group Identity

Ethnic and national identities were measured in Moroccan-origin adolescents with four questions. They indicated their identification with and valuation of their ethnic group (Moroccan) through two questions: “To what degree do you feel Moroccan?” and “how do you value being Moroccan?” (ethnic identity, *r* = 0.63, *p* < 0.001). They were also asked about their identification with and valuation of the host country with two questions: “To what degree do you feel Spanish?” and “how do you value being Spanish?” (national identity, *r* = 0.70, *p* < 0.001). Average scores in both questions (for each identity) were obtained.

#### Socio-Demographic Variables

Participants reported their sex, age, country of birth, country of birth of parents (father and mother), and age of arrival in Spain (for Moroccan participants). The questionnaire included more variables since it was part of a broader national project but only those relevant for the objectives of this study are reported.

### Procedure

Analysis of population registers (INE, 2019) was carried out to determine the number, geographical distribution, and countries of origin of foreign adolescents living in Spain. Subsequently, public secondary schools with a high number of adolescents of Moroccan origin were selected in provinces with a high presence of this population. Schools were contacted and the appropriate permits were requested to carry out the study. The parents/legal guardians of the adolescents signed an informed consent form that was collected at the time of application of the questionnaire. Teachers, families, and adolescents were informed of the objectives of the research, the people in charge, their voluntary participation, and the possibility of stopping at any time. Likewise, they were informed that the data collected would be treated anonymously and confidentially. The questionnaires were completed in paper and pencil format, and applied by trained personnel, in the classrooms of 16 public secondary schools of five Spanish provinces. The study was approved by the Human Research Bioethics Committee of the researchers’ university. The database used in this research has been made publicly available and can be accessed at Open Science Framework (OSF) https://osf.io/khmvu/?view_only=f41e41bf48a349c689469481baf63eaf.

## Results

### Preliminary Analyses

Confirmatory factor analysis was conducted to test the fit of the four-factor stereotypes model, which included the dimensions of morality, immorality, sociability, and competence. In addition, the fit of the two-factor model (warmth and competence; Fiske et al., 2002) and the three-factor model (morality sociability, and competence; Leach et al., 2007) were checked out. We used the chi-square test and calculated the root mean square error approximation (RMSEA), the comparative fit index (CFI), and the Tuker-Lewis index (TLI) to test the fit of these models. For general interpretation, models with RMSEA values greater than 0.10 and CFI and TLI values below 0.90 should be rejected (Brown, 2015). We also report the Akaike information criterion (AIC) and the Bayesian information criterion (BIC). Smaller values of these indices indicate better model fit. The maximum likelihood (ML) method was used to estimate the parameters and correlation between the factors were freed.

The four-factor stereotypes model showed a good fit of the data for both groups: Spanish adolescents, $\chi^{2}$ (113) = 278.05, *p* < 0.001, CFI = 0.95, TLI = 0.95, RMSEA = 0.05, AIC = 19863.10, BIC = 20030.13; and Moroccan-origin adolescents, $\chi^{2}$ (113) = 191.42, *p* < 0.001, CFI = 0.94, TLI = 0.93, RMSEA = 0.04, AIC = 15489.01, BIC = 15642.63. All factor loadings were statistically significant (see Figure 1). Results of the CFA for the three-factor and two-factor models for both groups indicated that they should be discarded: Three-factor model for Spanish adolescents, $\chi^{2}$ (116) = 500.32, *p* < 0.001, CFI = 0.90, TLI = 0.88, RMSEA = 0.08, AIC = 20209.63, BIC = 20364.37; and for Moroccan-origin adolescents, $\chi^{2}$ (116) = 325.98, *p* < 0.001, CFI = 0.85, TLI = 0.83, RMSEA = 0.07, AIC = 15778.15, BIC = 15920.68; Two-factor model for Spanish adolescents, $\chi^{2}$ (118) = 554.22, *p* < 0.001, CFI = 0.88, TLI = 0.86, RMSEA = 0.09, AIC = 20259.53, BIC = 20405.90; and for Moroccan-origin adolescents, $\chi^{2}$ (118) = 440.03, *p* < 0.001, CFI = 0.77, TLI = 0.74, RMSEA = 0.09, AIC = 1588.21, BIC = 16023.03.

Descriptive statistics and bi-variate Pearson correlations among variables for both Spanish and Moroccan-origin adolescents are reported in Table 2.

**Table 2:** Correlations among variables for Spanish and Moroccan-origin adolescents.

	1	2	3	4	5	6	7	8	9	10	Spanish adolescents		Moroccan-origin adolescents	
											*M*	*SD*	*M*	*SD*
1. Maintenance		0.08	0.05	0.01	-	-	0.19^**^	−0.06	0.18^**^	0.22^**^	3.57	0.60	3.83	0.71
2. Adoption	0.11^*^		0.34^**^	0.09^*^	-	-	0.49^**^	−0.29^**^	0.47^**^	0.28^**^	2.78	0.72	3.31	0.73
3. Preference for maintenance	0.62^**^	0.11^**^		−0.03	-	-	0.33^**^	−0.17^**^	0.38^**^	0.28^**^	3.11	0.92	3.85	0.85
4. Preference for adoption	0.08	0.60^**^	0.20^**^		-	-	−0.01	0.11^*^	0.01	0.01	3.44	0.91	3.31	0.83
5. Ethnic identity	0.36^**^	−0.05	0.34^**^	−0.12^*^		-	-	-	-	-	-	-	4.37	0.79
6. National identity	−0.04	0.11^*^	0.02	0.26^**^	−0.19^**^		-	-	-	-	-	-	3.06	1.11
7. Morality	0.13^*^	0.24^**^	0.13^*^	0.23^**^	−0.01	0.07		−0.58^**^	0.80^**^	0.59^**^	2.77	0.79	3.28	0.70
8. Immorality	0.01	−0.06	−0.06	−0.20^**^	0.08	−0.06	−0.43^**^		−0.54^**^	−0.40^**^	2.98	0.79	2.60	0.82
9. Sociability	0.06	0.22^**^	0.12^*^	0.22^**^	−0.04	0.17^**^	0.53^**^	−0.32^**^		0.61^**^	3.04	0.85	3.66	0.74
10. Competence	0.09	0.14^**^	0.16^**^	0.18^**^	−0.01	0.11^*^	0.46^**^	−0.25^**^	0.49^**^		3.26	0.74	3.50	0.66

Correlations for Spanish adolescents are reported above diagonal; correlations for Moroccan-origin adolescents are reported below diagonal.

** *p* < 0.01;

* *p* < 0.05.

### Intergroup Differences in Stereotypes

In order to compare intergroup stereotypes and acculturation perceptions and preferences, two multivariate analysis of variances (MANOVAs) were performed: stereotype dimensions and acculturation perceptions and preferences were included as dependent variables and the target/group (Moroccan youth evaluated by Spanish adolescents vs. Spanish youth evaluated by adolescents of Moroccan origin) as independent variable.

**Figure 2:**
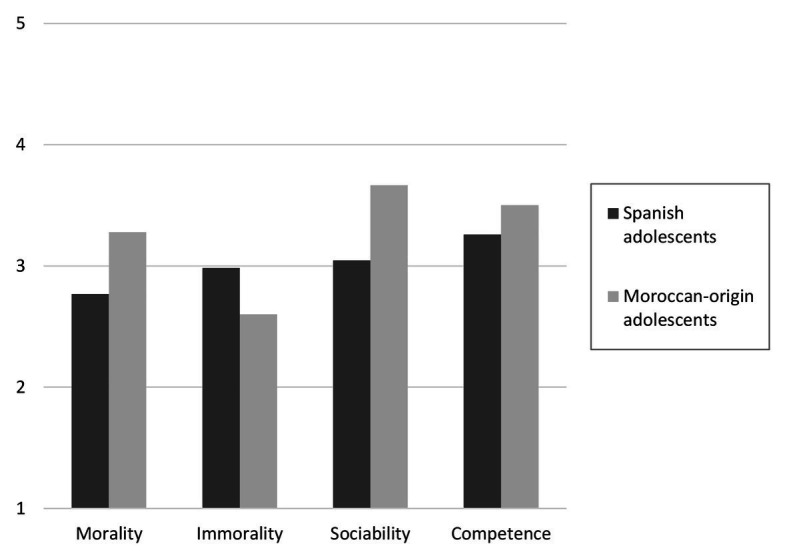
Intergroup perceptions of Spanish and Moroccan-origin adolescents in the four stereotype dimensions.

Results of the MANOVA showed that there was a multivariate effect of the group in the stereotype dimensions as combined dependent variables, Wilk’s *λ* = 0.86, *F*(4, 820) = 33.37, *p* < 0.001, *η*^2^_p_ = 0.14. As shown in Figure 2 (see also means and SDs in Table 2), adolescents of Moroccan origin had a more positive perception of Spanish youth compared to the perception that Spanish adolescents had of Moroccan youth (H1). Statistically significant differences appeared in all four stereotype dimensions analyzed. Moroccan youth were perceived by Spanish adolescents as less moral, *F*(1,823) = 91.72, *p* < 0.001, *η*^2^_p_ = 0.10; more immoral, *F*(1,823) = 45.49, *p* < 0.001, *η*^2^_p_ = 0.05; less sociable, *F*(1,823) = 119.32, *p* < 0.001, *η*^2^_p_ = 0.13; and less competent, *F*(1,823) = 23.44, *p* < 0.001, *η*^2^_p_ = 0.03, compared to the perception that Moroccan-origin adolescents had about Spanish youth. Homogeneity of variances for between-subjects comparison was not assumed. However, *t*-tests with corrections revealed the same results.

### Intergroup Differences in Acculturation Orientations

**Figure 3:**
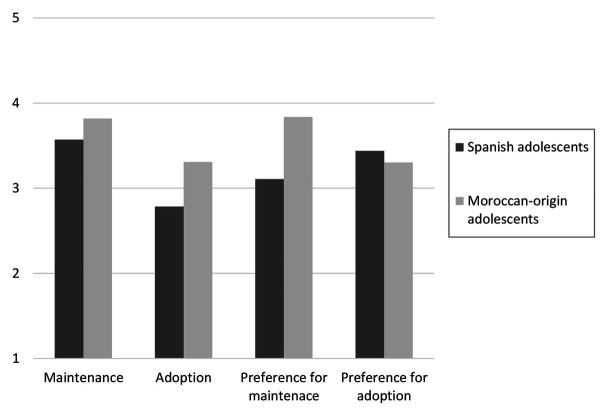
Acculturation orientations of Spanish and Moroccan-origin adolescents.

Results of the MANOVA showed that there was a multivariate effect of the group in the acculturation perceptions and preferences as combined dependent variables, Wilk’s *λ* = 0.78, *F*(4, 807) = 54.65, *p* < 0.001, *η*^2^_p_ = 0.21. As shown in Figure 3, there were some discrepancies between what Spanish adolescents perceived that Moroccan youth were doing and what Moroccan-origin adolescents admitted doing (H2a). Spanish adolescents perceived that Moroccan youth were adopting less than they claimed to do, *F*(1,810) = 103.10, *p* < 0.001, *η*^2^_p_ = 0.11; but also maintaining less than they claimed to do, *F*(1,810) = 28.71, *p* < 0.001, *η*^2^_p_ = 0.03. Regarding their preferences, in line with H2b, Spanish adolescents preferred Moroccan youth to maintain less than Moroccan-origin adolescents wanted to do, *F*(1,810) = 132.33, *p* < 0.001, *η*^2^_p_ = 0.14; and preferred more adoption than the latter desired to do, *F*(1,810) = 4.79, *p* = 0.029, *η*^2^_p_ = 0.01.

### Preference for Adopting the Host Culture

Four multiple linear regression analyses were conducted to analyze the effect of stereotypes in acculturation preferences (two multiple linear regressions for each group: one for preferences for maintenance and one for preferences for adoption). In order to control the effect of acculturation perceptions (for both groups) and ethnic and national identity (only for Moroccan-origin adolescents), these variables were included in Step 1. In Step 2, stereotype dimensions were added.

For adolescents of Moroccan origin, the results of the multiple regression analysis are shown in Table 3. Adopting the host culture and national identity were positively related to the desire to adopt the Spanish culture in Step 1. There was a statistically significant increase in the explained variance (∆*R*^2^ = 0.019) in Step 2. Interestingly, perceiving Spanish youth as immoral (e.g., aggressive, malicious, and false) significantly contributed to the model. The more immoral Spanish youth were perceived, the less willingness to adopt the Spanish culture.

**Table 3:** Multiple regression analysis for predicting preference for adoption among Moroccan-origin adolescents.

		Preference for adoption of Moroccan-origin adolescents				
		*B*	*SE*	*β*	*t*	*p*
1	Intercept	0.92	0.31		2.96	0.003
	Maintenance	0.04	0.05	0.04	0.78	0.439
	**Adoption**	**0.64**	**0.05**	**0.56**	**12.56**	**< 0.001**
	Ethnic identity	−0.08	0.05	−0.08	−1.60	0.110
	**National identity**	**0.14**	**0.03**	**0.19**	**4.27**	**< 0.001**
*F*(4,318) = 51.97, *p* < 0.001, *R*^2^_adjusted_ = 0.388						
2	Intercept	1.10	0.40		2.74	0.007
	Maintenance	0.04	0.05	0.03	0.70	0.487
	**Adoption**	**0.62**	**0.05**	**0.54**	**11.90**	**< 0.001**
	Ethnic identity	−0.07	0.05	−0.07	−1.43	0.155
	**National identity**	**0.14**	**0.03**	**0.18**	**4.02**	**< 0.001**
	Morality	0.01	0.07	0.01	0.10	0.918
	**Immorality**	**−0.12**	**0.05**	**−0.12**	**−2.49**	**0.013**
	Sociability	0.03	0.06	0.02	0.42	0.676
	Competence	0.03	0.06	0.02	0.41	0.678
*F*(8,314) = 27.81, *p* < 0.001; ∆*R*^2^ = 0.019, *p* = 0.036						

The results of significant predictors are in bold type.

For Spanish adolescents, results of the multiple regression analysis are shown in Table 4. Only perception of adoption of the host culture was marginally related to their preference for Moroccan youth to adopt the Spanish culture in Step 1. There was a statistically significant increase in the explained variance (∆*R*^2^ = 0.021) in Step 2. Perceiving Moroccan youth as immoral (e.g., aggressive, malicious, and false) also contributed to the model. The more immoral Moroccan youth were perceived, the more the preference for them to adopt the Spanish culture. These findings suggest that perceived immorality might be important for understanding preference for adopting the host culture for both majority and minority groups.

**Table 4:** Multiple regression analysis for predicting preference for adoption among Spanish adolescents.

		Preference for adoption of Spanish adolescents				
		*B*	*SE*	*β*	*t*	*p*
1	Intercept	3.23	0.29		11.04	< 0.001
	Perceived maintenance	−0.03	0.07	−0.02	−0.43	0.852
	**Perceived adoption**	**0.11**	**0.06**	**0.09**	**1.90**	**0.059**
*F*(2,470) = 1.84, *p* = 0.160, *R*^2^_adjusted_ = 0.004						
2	Intercept	2.41	0.43		5.65	< 0.001
	Perceived maintenance	−0.04	0.07	−0.02	−0.51	0.610
	**Perceived adoption**	**0.15**	**0.07**	**0.12**	**2.17**	**0.031**
	Morality	−0.04	0.10	−0.03	−0.40	0.690
	**Immorality**	**0.18**	**0.07**	**0.15**	**2.70**	**0.007**
	Sociability	0.05	0.09	0.05	0.58	0.561
	Competence	0.05	0.07	0.04	0.70	0.482
*F*(6,466) = 2.19, *p* = 0.043; ∆*R*^2^ = 0.021, *p* = 0.054						

The results of significant predictors are in bold type.

#### The Moderating Role of Stereotypes in Discrepancies of Preference for Adoption

To verify if stereotypes can moderate the discrepancies between majority and minority members in their acculturation preferences, we conducted four multiple regression analyses with the macro PROCESS 3.5 for SPSS, Model 1 (Hayes, 2018) using a bias-corrected bootstrap of 5,000 samples. Group was defined each time as predictor (1 = Spanish adolescents; 0 = Moroccan-origin adolescents) of preference for adoption and each stereotype dimension was defined as moderator (+*SD*, Mean, and −*SD*). The other stereotype dimensions were controlled as covariates. A heteroscedasticity consistent standard error and covariance matrix estimator was used.

**Figure 4:**
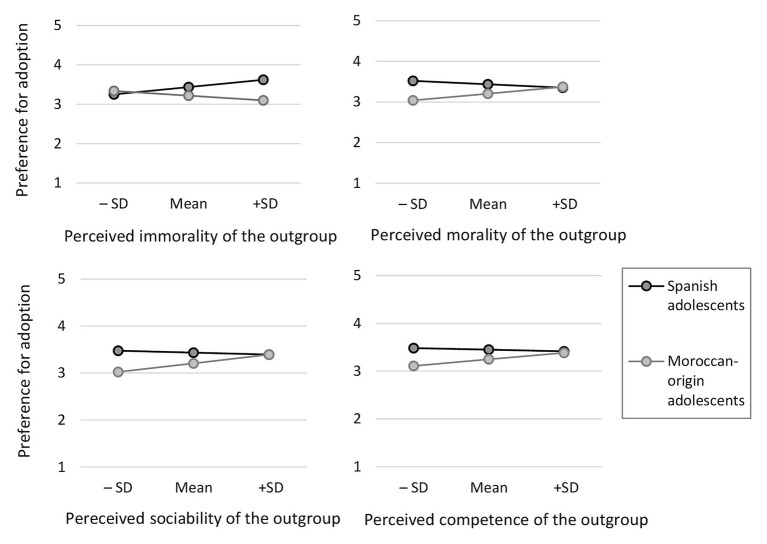
Two-way interaction between intergroup stereotypes and group when predicting preference for adopting the host culture.

The regression analyses yielded a significant interaction between group and perceived immorality, *B* = 0.37, *SE* = 0.08, *t*(802) = 4.62, ∆*R*^2^ = 0.027, *p* < 0.001, 95% CI [0.213, 0.527]; perceived morality, *B* = −0.32, *SE* = 0.09, *t*(802) = −3.50, ∆*R*^2^ = 0.016, *p* < 0.001, 95% CI [−0.494, −0.139]; perceived sociability, *B* = −0.26, *SE* = 0.09, *t*(802) = −2.99, ∆*R*^2^ = 0.013, *p* = 0.003, 95% CI [−0.437, −0.091]; and perceived competence, *B* = −0.24, *SE* = 0.10, *t*(802) = −2.43, ∆*R*^2^ = 0.008, *p* = 0.015, 95% CI [−0.429, −0.045]. The negative two-way interactions mean that the discrepancy between majority and minority adolescents in their preferences for minority groups to adopt the host culture decreases as the level of perceived morality, sociability, and competence increases. The positive two-way interaction means the opposite, that the discrepancy decreases as the level of perceived immorality decreases (see Figure 4).

As shown in Figure 4, Spanish and Moroccan-origin adolescents showed strong discrepancies in their adoption preferences when the stereotypes of the other group were more negative. That is, when the other group was perceived as highly immoral (+*SD*), *B* = 0.53, *SE* = 0.10, *t*(802) = 5.08, *p* < 0.001, 95% CI [0.322, 0.728]; lowly moral (−*SD*), *B* = 0.48, *SE* = 0.11, *t*(802) = 4.27, *p* < 0.001, 95% CI [0.260, 0.702]; lowly sociable (−*SD*), *B* = 0.45, *SE* = 0.12, *t*(802) = 3.89, *p* < 0.001, 95% CI [0.224, 0.681]; or lowly competent (−*SD*), *B* = 0.37, *SE* = 0.11, *t*(802) = 3.50, *p* < 0.001, 95% CI [0.164, 0.581]. There were also differences in their adoption preferences with medium perceptions in immorality, *B* = 0.22, *SE* = 0.07, *t*(802) = 3.23, *p* = 0.001, 95% CI [0.087, 0.356]; morality, *B* = 0.23, *SE* = 0.07, *t*(802) = 3.28, *p* = 0.001, 95% CI [0.092, 0.368]; sociability, *B* = 0.23, *SE* = 0.07, *t*(802) = 3.20, *p* = 0.001, 95% CI [0.087, 0.364]; or competence, *B* = 0.20, *SE* = 0.07, *t*(802) = 2.92, *p* = 0.004, 95% CI [0.067, 0.339]. However, their preferences in adoption totally matched (i.e., there were no discrepancies) when the intergroup perception is highly positive. That is, when the other group was perceived with the lowest levels of immorality (−*SD*), *B* = −0.08, *SE* = 0.09, *t*(802) = −0.97, *p* = 0.333, 95% CI [−0.251, 0.085]; and the highest levels of morality (+*SD*), *B* = −0.02, *SE* = 0.09, *t*(802) = −0.25, *p* = 0.805, 95% CI [−0.190, 0.148]; sociability (+*SD*), *B* = −0.01, *SE* = 0.09, *t*(802) = −0.02, *p* = 0.984, 95% CI [−0.177, 0.173]; or competence (+*SD*), *B* = 0.03, *SE* = 0.09, *t*(802) = 0.36, *p* = 0.716, 95% CI [−0.144, 0.209].

#### Two-Way Interaction Between Perceived Adoption and Stereotypes

**Figure 5:**
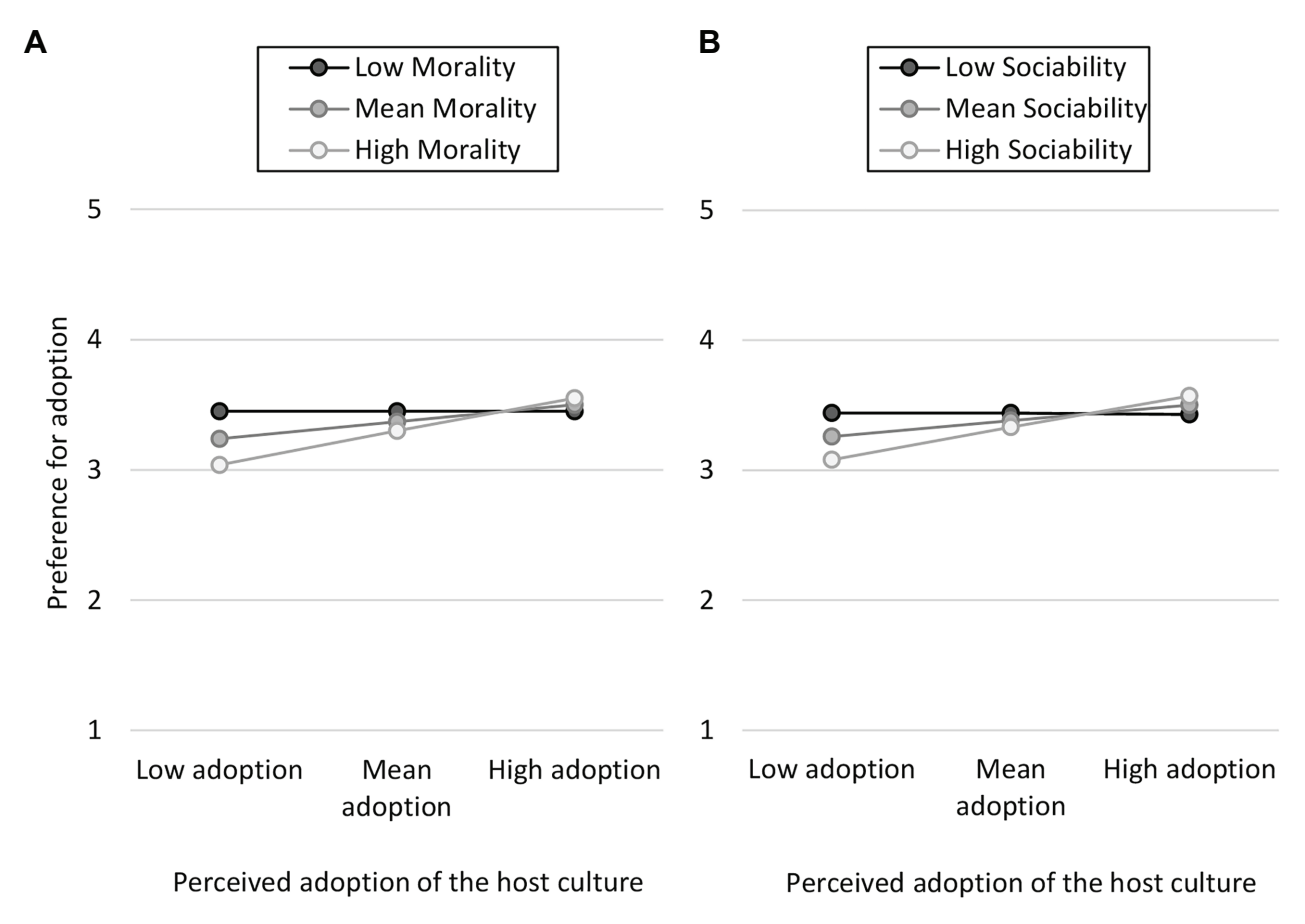
Two-way interaction between morality **(A)** and sociability **(B)** stereotypes and perceived adoption when predicting preference for adopting the host culture.

A multiple regression analysis using the macro PROCESS (Hayes, 2018) tested if the relation between stereotypes (X) and preference for adoption (Y) could be conditioned by perceived adoption (W) in majority members in order to test the fourth hypothesis. A two-way interaction with perceived morality was significant, *B* = 0.22, *SE* = 0.07, *t*(475) = 3.15, ∆*R*^2^ = 0.025, *p* = 0.002, CI [0.083, 0.358]. This positive two-way interaction means that the relation between perceived morality and preference for adoption is increasingly negative as the level of perceived adoption decreases. As shown in Figure 5 (A), only when Spanish participants perceived that Moroccan youth were not adopting the host culture, perceived morality was negatively associated to preference for adoption, *B* = −0.26, *SE* = 0.08, *t*(475) = −3.04, *p* = 0.003, CI [−0.422, −0.090].

There was also a two-way interaction with perceived sociability with the same pattern, *B* = 0.21, *SE* = 0.07, *t*(475) = 3.04, ∆*R*^2^ = 0.025, *p* = 0.003, CI [0.073, 0.339]. This positive two-way interaction means that the relation between perceived sociability and preference for adoption is increasingly negative as the level of perceived adoption decreases. As shown in Figure 5 (B), only when Spanish participants perceived that Moroccan youth were not adopting the host culture, perceived sociability was negatively associated to preference for adoption, *B* = −0.21, *SE* = 0.09, *t*(475) = −2.45, *p* = 0.015, [−0.385, −0.042]. To summarize, when Spanish adolescents perceived that Moroccan youth were not adopting the Spanish culture, perceived morality and sociability played a role in their acculturation preferences regarding adoption. The less moral and sociable Moroccans were perceived, the more preference for adoption. No other interactions were significant.

### Preference for Maintaining the Origin Culture

For Moroccan-origin adolescents, results of the multiple regression analysis are shown in Table 5. Maintenance and ethnic identity were positively related to preference for maintenance. Step 2 showed that stereotypes about Spanish youth did not contribute to the model.

**Table 5:** Multiple regression analysis for predicting preference for maintenance among Moroccan-origin adolescents.

		Preference for maintenance of Moroccan-origin adolescents				
		*B*	*SE*	*β*	*t*	*p*
1	Intercept	0.30	0.32		0.93	0.352
	**Maintenance**	0.**67**	**0.06**	**0.56**	**12.00**	**< 0.001**
	Adoption	0.05	0.05	0.04	0.97	0.334
	**Ethnic identity**	**0.16**	**0.05**	**0.15**	**3.07**	**0.002**
	National identity	0.05	0.03	0.06	1.34	0.181
*F*(4,321) = 53.84, *p* < 0.001, *R*^2^_adjusted_ = 0.394						
2	Intercept	0.12	0.41		0.28	0.776
	**Maintenance**	**0.67**	**0.06**	**0.56**	**11.82**	**< 0.001**
	Adoption	0.03	0.05	0.03	0.61	0.541
	**Ethnic identity**	**0.16**	**0.05**	**0.15**	**3.12**	**0.002**
	National identity	0.04	0.03	0.05	1.04	0.300
	Morality	−0.04	0.07	−0.03	−0.58	0.565
	Immorality	−0.05	0.05	−0.04	−0.81	0.366
	Sociability	0.03	0.06	0.03	0.51	0.608
	Competence	0.12	0.07	0.09	1.75	0.081
*F*(8,317) = 27.90, *p* < 0.001; ∆*R*^2^ = 0.012, *p* = 0.180						

The results of significant predictors are in bold type.

For Spanish adolescents, the results of the multiple regression analysis are shown in Table 6. In Step 1, perception of adoption of the host culture was positively related to preference for maintaining the origin culture among Spanish adolescents. There was a statistically significant increase in the explained variance (∆*R*^2^ = 0.021) in Step 2, showing that perceived sociability contributed to the model. The more sociable Moroccan youth are perceived, the more preference for them to conserve their original culture.

**Table 6:** Multiple regression analysis for predicting preference for maintenance among Spanish adolescents.

		Preference for maintenance of Spanish adolescents				
		*B*	*SE*	**β**	*t*	*p*
1	Intercept	1.69	0.28		6.16	0.000
	Perceived maintenance	0.05	0.07	0.03	0.78	0.436
	**Perceived Adoption**	**0.45**	**0.05**	**0.35**	**8.13**	**< 0.001**
*F*(2,469) = 33.95, *p* < 0.001, *R*^2^_adjusted_ = 0.123						
2	Intercept	0.95	0.39		2.44	0.015
	Perceived maintenance	−0.03	0.07	−0.02	−0.45	0.654
	**Perceived adoption**	**0.29**	**0.06**	**0.23**	**4.72**	**< 0.001**
	Morality	0.03	0.09	0.03	0.35	0.726
	Immorality	0.09	0.06	0.08	1.46	0.144
	**Sociability**	**0.24**	**0.08**	**0.23**	**3.05**	**0.002**
	Competence	0.11	0.07	0.09	1.69	0.093
*F*(6,465) = 17.68, *p* < 0.001; ∆*R*^2^ = 0.06, *p* < 0.001						

The results of significant predictors are in bold type.

Perceived immorality and perceived competence did not moderate the discrepancies between majority and minority groups in their maintenance preferences. Perceived morality did it marginally, *B* = 0.17, *SE* = 0.09, *t*(806) = 1.93, ∆*R*^2^ = 0.004, *p* = 0.054, 95% CI [−0.003, 0.333]. Only perceived sociability significantly reduced these differences, *B* = 0.23, *SE* = 0.08, *t*(806) = 2.91, ∆*R*^2^ = 0.008, *p* = 0.004, 95% CI [0.076, 0.389]; but in any case, these discrepancies disappeared, as Moroccan-origin adolescents always prefer more maintenance than what Spanish adolescents wanted independently of the perceived sociability of the other group, *p* < 0.001. Stereotypes did not interact with perceived adoption or perceived maintenance for the preference for maintenance.

## Discussion and Conclusions

The main question that motivated this research was to understand the specific role that distinct stereotype dimensions play in the acculturation preferences of Spanish adolescents and adolescents of Moroccan-origin living in Spain. We analyzed the mutual stereotypes (Objective 1), and the majority-minority discrepancies in adolescents’ acculturation perceptions and preferences (Objective 2), the specific contribution of four different stereotype dimensions (i.e., morality, immorality, sociability, and competence) to acculturation preferences (Objective 3), whether stereotypes could moderate the discrepancies between Spanish adolescents and adolescents of Moroccan-origin in their acculturation preferences (Objective 4), and whether stereotypes might be more predictive of majority’s acculturation preferences under more threatening circumstances such as when they perceive minorities are not adopting the host culture (Objective 5).

The results confirmed, as it was expected, that Spanish adolescents evaluated Moroccan youth more negatively than adolescents of Moroccan origin evaluated their Spanish peers, confirming Hypothesis 1. The devalued group members, even if they are discriminated against, maintain a relatively positive perception of the majority group members. This finding is not surprising giving the status of these social groups in Spanish society. On the one hand, the mainstream culture in Spain does not include a wide cultural diversity, being the traditional Spanish culture the norm. Norms establish what is more valuable in society. Therefore, it is not strange that Spanish adolescents are more positively assessed by their immigrant-origin peers who presumable share mainstream norms. On the other hand, all cultural groups associated to Islam have negative connotations in Spain, being Moroccans a traditionally stigmatized group in this country, judged as less moral and more threatening immigrants than other groups (Navas et al., 2012; López-Rodríguez et al., 2013). These findings may have some relevant implications in terms of collective actions for inequality. Ethnic minority groups who maintain positive relations with the majority group tend to express positive attitudes toward the dominant group, something that could be associated to less anger toward inequality and less collective action to change the *statu quo* (Tausch et al., 2015).

Regarding the intergroup discrepancies on acculturation orientations, Spanish adolescents perceived that Moroccan youth were adopting less than the adolescents of Moroccan origin claimed to do, but they also perceived that Moroccan youth were maintaining less than Moroccan adolescents claimed to do. So, Hypothesis 2a is only partially met. However, these findings are in line with previous research in which it was found that immigrant adolescents living in Spain claimed that they maintain and adopt more compared to what Spanish adolescents perceived (López-Rodríguez et al., 2014a; Mancini et al., 2018). In the Hypothesis 2b, we expected that Spanish adolescents would prefer Moroccan youth to adopt more and maintain less than what they would desire for themselves (H2b). This hypothesis was confirmed and our results are similar to those found in the adult population applying the RAEM (Navas et al., 2005, 2007; Navas and Rojas, 2010). They diverge slightly compared to previous research with adolescents in Spain that found that immigrant adolescents preferred to maintain, but also to adopt more compared to what Spanish adolescents wanted (López-Rodríguez et al., 2014a; Mancini et al., 2018). However, this previous research included immigrants from different ethnic origins in the analyses, whereas the present research considered only adolescents of Moroccan origin. As stated before, Moroccans are a highly stigmatized group in Spain compared to other immigrants from different origins. The pattern found in this study is coherent to a relatively threatening perception of adolescents of Moroccan-origin, as Spanish adolescents demanded less maintenance of the Moroccan culture and more adoption of the Spanish culture than what this minority group prefers to do. However, it is fair to recognize that those discrepancies in their preference for adoption were smaller than their discrepancies in preference for maintenance.

Regarding Hypothesis 3, it was confirmed that stereotypes (especially the dimension of immorality) played an important role in understanding preference for cultural adoption of Spanish and Moroccan-origin adolescents beyond acculturation perceptions (in Spanish-origin adolescents) and acculturation strategies and national identity (in Moroccan-origin adolescents). For participants from the minority group, the extent to which they are already adopting the host culture and the level of national identity were positively related to the desire of adopting the Spanish culture. Interestingly, perceiving Spanish youth as immoral (e.g., aggressive, malicious, and false) significantly contributed to the model, indicating that the more immoral Spanish youth are perceived, the less the desire of adopting the Spanish culture. Likewise, for Spanish participants, perceiving Moroccan youth as immoral also contributed to the model. The more immoral Moroccan youth were perceived, the higher the Spaniards’ preference for Moroccan youth to adopt the Spanish culture. Three important conclusions are derived from these findings. The first one is related to the ineludible interactive nature of acculturation: perceived immorality was important for both minority and majority groups when understanding their acculturation preferences but in the opposite direction. The immoral character of the outgroup was negatively related to preference for adoption among Moroccan-origin adolescents but positively related to preference for adoption among Spanish adolescents. This fact reveals that mutual stereotypes are important from both perspectives. The second conclusion stands on the importance of stereotypes especially for preference for adopting the host culture. Adoption of the host culture is important because it connects to social integration with the other group. From the minority perspective, if we do not trust the other, it has no sense to approach their culture. From the majority perspective, if we do not trust the other, demanding adoption of the host culture might neutralize the threat that implies their immoral character. This is coherent to previous research (e.g., Zick et al., 2001; Arends-Tóth and Van de Vijver, 2003) that has found that majority members preferred that minority members adopted the host culture instead of maintaining the origin culture. The final conclusion is that immorality seems especially diagnostic in the accommodating process of acculturation, as it does in the impression-formation process (e.g., Skowronski and Carlston, 1987), probably due to the evolutionary social function that these traits comply and their relation to threat. Thus, perceived immorality was an important stereotypical dimension to consider for understanding the preference for adopting the host culture for both majority and minority groups, and its power in intergroup relations was confirmed (Sayans-Jiménez et al., 2017).

The relation between intergroup stereotypes and acculturation preferences, however, might be more complex than it seems. Although there is only a direct relationship between perceived immorality and preference for adoption in both groups, the four stereotype dimensions can also modulate the discrepancies between majority and minority groups regarding their adoption preferences (H4). Stereotypes, these social keys to guide judgments in complex situations, can harsh or soften intergroup discrepancies in adoption preferences. It is precisely when the other group is perceived as highly immoral, or lowly moral, social, and competent when Spanish adolescents’ and Moroccan-origin adolescents’ adoption preferences separate more. On the contrary, these discrepancies disappear when the other is perceived to be a source of trust, kindness, and competence. This new way to understand the role of stereotypes in the acculturation process might offer new possibilities for intervention and cultural understanding. As far as we know, no previous evidence has shown that stereotypes could increase/decrease acculturation discrepancies between different cultural groups. The way we see others can be a potential tool to come together and join different acculturation perspectives.

However, stereotypes might not be always useful in the decisions of majority members about acculturation. In an attempt to understand how and when stereotypes relate to the acculturation preferences of majority members about how minorities should acculturate, we analyzed whether their stereotypes could interact with other factors such as their own acculturation perceptions (H5). In this study, stereotypes of morality and sociability interacted with perceived adoption when predicting preference for adoption. When Spanish adolescents perceived that Moroccan youth were not adopting the Spanish culture, perceived morality and sociability played a role in their preferences for adoption. The less moral and sociable Moroccan youth were perceived, the more preference for cultural adoption. This result is in line with previous research that demonstrates the relevant role of the degree of perceived adoption (over perceived maintenance) from the majority perspective (López-Rodríguez et al., 2014b). We believe that it is due to the fact that when people perceive that “others” do not want to adopt the host culture, the host population support more cultural adoption (and not cultural maintenance) in order to assimilate them, especially if the outgroup is perceived as a threat (Tip et al., 2012; Stephan et al., 2016) or with low morality like in our study. To perceive a group as low in morality is to be able to identify a potential threat to the in-group (Brambilla et al., 2011, 2012; Brambilla and Leach, 2014). Thus, morality (and in this case, also sociability) seems to neutralize the perceived threat that a low perceived adoption can provoke in members of the majority group. Future research should clarify additional conditions under which stereotypes might be more strongly related to acculturation preferences.

An important limitation of the present research is that it was correlational and hence did not allow inferences about causality. Indeed, it is plausible to suppose that intergroup stereotypes determine acculturation preferences and vice versa. People can use stereotypes with a justifying function of their preferences for acculturation (i.e., we perceive them as less moral – or immoral – because “they do not want to adopt our culture or follow our customs and norms”). Experimental studies would corroborate the proposed direction of our results and contrast the alternative roles that stereotypes might play when understanding preferences for adopting the host culture. We can also consider that the variance explained by the stereotypes was limited. Intergroup stereotypes are not, by themselves, the most important factor when trying to understand acculturation preferences. However, they contribute and can interact with other factors such as acculturation perceptions. Future research should explore in more detail the exact role that stereotypes, especially perceived immorality, play in acculturation preferences and discard alternative hypotheses.

Despite the potential improvements that could be made, we consider that these results contribute to the psychosocial literature on acculturation and stereotypes adding evidence to the need for a more context-situated analysis of the acculturation processes of adolescent ethnic minorities and the importance of considering stereotypes in those processes, especially the attributions of immorality to the outgroup. Moreover, results of research connecting stereotypes and acculturation processes with adolescents have additional implications, since adolescence is a period in which consequences of non-harmonic acculturation processes can have more negative effects and lead to problems of psychological adaptation more easily than in adulthood (Schwartz et al., 2006). In terms of intervention, understanding these patterns in adolescence facilitates the change of stereotypes and biased intergroup perceptions before they get firmly established in adulthood, so it can be highly efficient for improving intergroup relations in the future.

In conclusion, we believe that mutual perceptions and acculturation discrepancies between majority and minority groups can contribute to either soften or harden intercultural relations in the multicultural society that Europe is today. With the increase of migration flows and the coexistence of diverse cultural groups, the study of acculturation processes and involved psychosocial variables such as intergroup stereotypes are essential to understand intercultural relations, and to generate adequate psychological and political responses to support inclusive societies.

## Data Availability Statement

The datasets used for this study can be found in Open Science Framework (OSF) at: https://osf.io/khmvu/?view_only=f41e41bf48a349c689469481baf63eaf.

## Ethics Statement

The studies involving human participants were reviewed and approved by Bioethics Commission from University of Almería. Written informed consent to participate in this study was provided by the participants’ legal guardian/next of kin.

## Author Contributions

LL-R, AU, MN, MS-C, and IC developed the study concept and design, drafted the manuscript, provided critical revisions, and approved the final version of the manuscript for submission. Data collection was performed by MS-C and MN. MS-C, LL-R, and AU performed the data analysis. All authors contributed to the article and approved the submitted version.

### Conflict of Interest

The authors declare that the research was conducted in the absence of any commercial or financial relationships that could be construed as a potential conflict of interest.
